# Maternal Fructose Intake Exacerbates Cardiac Remodeling in Offspring with Ventricular Pressure Overload

**DOI:** 10.3390/nu13093267

**Published:** 2021-09-18

**Authors:** Steve Leu, Kay L. H. Wu, Wei-Chia Lee, You-Lin Tain, Julie Y. H. Chan

**Affiliations:** 1 Institute for Translational Research in Biomedicine, Kaohsiung Chang Gung Memorial Hospital, Kaohsiung 833401, Taiwan; wlh0701@yahoo.com.tw (K.L.H.W.); tainyl@hotmail.com (Y.-L.T.); 2 Department of Biotechnology, College of Life Science, Kaohsiung Medical University, Kaohsiung 807378, Taiwan; 3 Department of Urology, Kaohsiung Chang Gung Memorial Hospital and Chang Gung University College of Medicine, Kaohsiung 833401, Taiwan; dinor666@ms32.hinet.net; 4 Department of Pediatrics, Kaohsiung Chang Gung Memorial Hospital and Chang Gung University College of Medicine, Kaohsiung 833401, Taiwan

**Keywords:** maternal fructose exposure, developmental programming, cardiac hypertrophy, oxidative stress, transverse aortic constriction

## Abstract

Recent studies demonstrated that metabolic syndrome and cardiovascular diseases could be elicited by developmental programming, which is regulated by prenatal nutritional and environmental stress. In this study, we utilized a rat model to examine the effect of excessive maternal fructose intake during pregnancy and lactation on cardiac development and progression of pressure overload-induced cardiac hypertrophy in offspring. Transverse aortic constriction (TAC) was performed on 3-month-old male offspring to induce ventricular pressure overload. Four weeks post-TAC, echocardiographic assessment as well as histopathological and biochemical examinations were performed on the myocardium of the offspring. Echocardiographic and gross examinations showed that heart weight, interventricular septal thickness in diastole (IVD; d), and left ventricular posterior wall thickness in diastole (LVPW; d) were elevated in offspring with TAC and further increased by maternal fructose exposure (MFE). However, the left ventricular ejection function was not significantly affected. Myocardial histopathological examination revealed that the indices of fibrosis and oxidative stress were higher in offspring with MFE and TAC than those in animals receiving either treatment. Molecular examinations on the myocardium demonstrated an MFE-induced upregulation of p38-MAPK signaling. Next generation sequence (NGS) analysis indicated a modulation of the expression levels of several cardiac hypertrophy-associated genes, including GPR22, Myh7, Nppa, P2RX4, and Npy by MFE. Subsequent RT-PCR indicated that MFE regulated the expression levels of genes responsive to cardiac hypertrophy (i.e., Myh-7, ANP) and oxidative stress (i.e., GR, GPx, and NQO-1). In conclusion, MFE during pregnancy and lactation modulated myocardial gene expression, increased oxidative stress, and exacerbated ventricular pressure overload-induced cardiac remodeling in rat offspring.

## 1. Introduction

Previous studies have reported an adverse impact of the consumption of fructose-containing beverages, which has significantly increased during past decades [1], on metabolism and body adiposity [2,3] such as caloric overconsumption, body weight gain, and induction of metabolic syndromes [2,4,5]. Consistent with the findings of insulin resistance, hypertension, and cardiac function impairment in rats fed with a high fructose diet [4,6,7], clinical studies also reported an association of the intake of high quantities of fructose with tissue insulin insensitivity, metabolic defects, and development of a pre-diabetic state [8].

Early environmental and nutritional insults in the developmental stage may lead to permanent changes in tissue structure and function in adulthood, a concept now known as developmental programming [9]. Previous studies have demonstrated that heart function could be persistently affected by adverse hypoxia influences in utero [10,11,12]. On the other hand, other reports have shown that permanent impacts on myocardial function, especially ventricular hypertrophy, may be attributed to maternal undernutrition [13]. Maternal nutrient restriction and overnutrition in sheep models revealed changes of gene expression profiles in the fetal left ventricle accompanied by cardiac hypertrophy and fibrosis [14,15,16]. Clinical investigations further indicated that maternal obesity alters the cardiac function of offspring with an elevation in the risk of cardiovascular diseases [17], suggesting that prenatal programming may determine the level of vulnerability for heart disease before birth. The aforementioned studies indicated that an alternation in metabolic conditions during pregnancy may lead to adverse cardiac impacts from fetal cardiac developmental programming which could persist into adulthood.

Recent animal studies have demonstrated that excessive maternal fructose intake during pregnancy and lactation (i.e., maternal fructose exposure, MFE) contributes to hypertension and induces developmental programming in the kidney of offspring [18,19]. Our recent study also indicated an MFE-induced impairment of endothelial progenitor cells (EPC) angiogenic activity [20]. Moreover, offspring with MFE showed endothelial dysfunction ex vivo and poor injury recovery after critical limb ischemia [20]. All indicated that MFE might induce developmental programming and influence cardiovascular function in adulthood. However, the effects of MFE on cardiac developmental programming and myocardial stress response remain unknown.

In the present study, we applied a rat model with excessive maternal fructose consumption during pregnancy and lactation to investigate the role of MFE in cardiac developmental programming and susceptibility to cardiovascular diseases in offspring. Histopathological, biochemical, and echocardiographic examinations were performed to determine the cardiac structure, function, and stress response to ventricular pressure overload in offspring.

## 2. Materials and Methods

### 2.1. Animals and Transverse Aortic Constriction Operation

All animal experiments in this study were performed with the approval of the Institutional Animal Care and Use Committee of Chang Gung Memorial Hospital (No. 2015122209). For the experimental groups, a standard laboratory rat chow or a 60% high-fructose diet (Harlan Laboratories, Hayward, CA, USA) was provided for the mother rats immediately following mating till postnatal day 21, when the offspring were separated into groups by litter. Thirty-two male offspring were categorized into four groups: Group NC (normal control, offspring from dams fed with normal diet), Group MFE (maternal fructose exposure, offspring from dams fed with high fructose diet), Group NC + TAC (offspring from dams fed with normal diet and subjected to transverse aortic constriction), group MFE + TAC (offspring from dams fed with high fructose diet and subjected to transverse aortic constriction), *n* = 8 for each group. To induce ventricular pressure overload, 3-month-old rat offspring were treated with transverse aortic constriction. Inhalation of 4% isoflurane in oxygen was used for induction of anesthesia, while 2% isoflurane was used for maintenance of anesthesia during surgery. Left-side thoracotomies were conducted to expose the transverse aorta under mechanical ventilation (Harvard Apparatus, Holliston, MA, USA). With an overlying blunted 22-gauge needle, the transverse aorta was banded with a 4-0 silk suture. The needle was then quickly removed to create a defined constriction. Sham operations were performed in which the transverse aorta had been exposed but not banded. For analgesia, buprenorphine (0.05 mg/kg) was provided subcutaneously every 12 h for 48 h post-thoracotomy. Euthanasia of animals was performed by heart removal under anesthesia with isoflurane inhalation.

### 2.2. Cardiac Functional Assessment by Echocardiography

Transthoracic echocardiographic examinations were performed in each group on day 28 after TAC using an echocardiographic system (Vevo 2100 imaging system, Visual Sonics, Toronto, ON, Canada). Inhalation of 4% isoflurane in oxygen was used for induction of anesthesia, while 2% isoflurane was used for maintenance of anesthesia during echocardiography. M-mode tracings of LV were obtained with the heart being imaged in a 2-dimensional mode in the short-axis at the level of the papillary muscle. Left ventricular internal dimensions (end-systolic diameter (LVID; s) and end-diastolic diameter (LVID; d)) were measured according to the American Society of Echocardiography leading-edge method using at least three consecutive cardiac cycles. The LV ejection fraction (LVEF) was calculated as follows: LVEF (%) = [(LVID; d^3^-LVID; s^3^)/LVID, d^3^] × 100.

### 2.3. Quantitative Reverse Transcription-Polymerase Chain Reaction

Real-time RT-PCR with the Applied Biosystems 7900 HT Sequence Detection System (Applied Biosystems, Waltham, MA, USA) and TaqMan^®^ Gene Expression Assay was used to quantitatively examine mRNA expressions in the left ventricular myocardium. Reverse transcription on 10 ng of total RNA was performed with TaqMan^®^ Universal PCR Master Mix, no AmpErase (Applied Biosystems, Waltham, MA, USA) and the respective TaqMan^®^ reagents for target genes. RT-PCR was carried out in a total volume of 20 µL reaction mixture according to the manufacture’s protocol. Amplifications were carried out as follows: 95 °C for 10 min, 40 cycles of 95 °C for 15 s, and 60 °C for 60 s. Samples were analyzed in triplicate replication. The mRNA levels were defined from the cycle threshold (Ct), the comparative Ct method, and normalization by the level of β-actin in each sample.

### 2.4. Western Blot

Tissues from left ventricular myocardium were used for protein extraction. Equal amounts (10–30 μg) of protein were loaded and separated by SDS-PAGE using 8–12% acrylamide gradients. Following electrophoresis, the separated proteins were transferred electrophoretically to a polyvinylidene difluoride (PVDF) membrane (Amersham Biosciences, Piscataway, NJ, USA). Membranes were incubated in blocking buffer (5% nonfat dry milk in T-TBS containing 0.05% Tween 20) overnight to block non-specific proteins. The membranes were incubated with primary antibodies against p38 (1:500, Abcam, Cambridge, MA, USA), phosphorylated p38 (1:500, Abcam, Cambridge, MA, USA), GPR22 (1:500, Invitrogen, Carlsbad, CA, USA), and β-actin (1:10,000, Millipore, Burlington, MA, USA) for 1 h at room temperature. Signals were detected with HRP-conjugated goat anti-mouse or goat anti-rabbit with ECL (Perkin Elmer, Waltham, MA, USA).

### 2.5. Histopathological Staining

Masson’s Trichrome (MTC) was used to analyze the extent of collagen synthesis and deposition, cardiac sections (6 µm) at 3 mm intervals according to the protocol provided by the manufacturer. Cryosections were first fixed in Bouin’s solution, followed by incubation in Weigert’s iron hematoxylin solution. Slides then were stained with Biebrich scarlet-acid fuchsin and aniline blue, followed by dehydration in ethanol and xylene. The collagen fibers were stained blue, while nuclei were stained black and myocardium was stained red. For hematoxylin and eosin (H&E) staining, cryosections were fixed with 10% buffered formalin and incubated with hematoxylin for nuclear staining, while cytoplasm was stained with eosin. Sections were mounted with a mounting medium after dehydration with ethanol. To measure fibrotic areas in myocardial sections, ten randomly selected HPF (high power fields) images from each MTC or H&E staining section were selected and analyzed with a software Image J (National Institutes of Health, Bethesda, MD, USA). To quantitate myocardial arterial muscularization, three randomly selected vessels (diameters > 100 μm) from left ventricular free wall of each myocardial section were analyzed using Image J to measure vessel diameter and media thickness.

### 2.6. Assessment of Oxiditive Stress in the Myocardium

Detection of oxidized protein in the myocardium was performed with OxyBlot and OxyIHC oxidized protein detection kit (MilliporeSigma, S7150 and S7450, St. Louis, MO, USA) according to the manufacturer’s instructions. For OxyBlot, DNPH (2,4-dinitrophenylhydrazine) derivatization was carried out on 6 μg of protein extracted form myocardium of offspring for 15 min. Proteins were separated in a 12% SDS-polyacrylamide gel after DNPH derivatization, followed by transferal to nitrocellulose membranes and incubation in the primary antibody solution (anti-dinitrophenol (DNP) 1:150) for 2 h. After incubation in the secondary antibody solution (1:300) for 1 h at room temperature, the washing procedure was repeated eight times within 40 min. Immunoreactive bands were visualized by enhanced chemiluminescence (ECL; Amersham Biosciences UK, Buckinghamshire, UK). For OxyIHC, deparaffinized and rehydrated myocardial sections were covered with antigen retrieval buffer and incubated in a steamer for 20 min. After incubation with DNPH solution for 30 min at room temperature, sections were incubated with primary antibody solution, followed by incubation with biotinylated secondary antibody for 30 min at room temperature. After incubation with streptavidin conjugated HRP, sections were colored with DAB-A/B mixture.

### 2.7. Culturing of Cardiac Cells and Immunofluorescent Staining

To detect GPR22 expression in cardiomyocytes and non-cardiomyocyte cells, ventricular myocardium from 2-day-old Sprague–Dawley rats was isolated and digested with collagenase (0.4 mg/mL) and pancreatin (0.6 mg/mL) in 116 mM NaCl, 20 mM HEPES (pH 7.35), 0.8 mM NaH_2_PO_4_, 5.6 mM glucose, 5.4 mM KCl, 0.8 mM MgSO_4_. Cells were recovered by centrifugation and then resuspended in plating medium (80% DMEM, 20% M199, 15% fetal bovine serum (FBS), 100 U/mL of penicillin and streptomycin) and plated on gelatin pre-coated coverslips. For immunofluorescent staining, cells on coverslips were fixed with 4% paraformaldehyde and then permeated with 0.5% Triton X-100. Coverslips were then incubated with antibodies against GPR22 (1:200, Invitrogen, Carlsbad, CA, USA) and Troponin I (1:1000, Millipore, Burlington, MA, USA) at 4 °C overnight, followed by incubation with Alex488 or Alex594-conjugated goat anti-mouse or rabbit IgG (Invitrogen, Carlsbad, CA, USA). Samples were examined under a fluorescent microscope (BX53, Olympus, Tokyo, Japan) after nuclear counterstaining with DAPI.

### 2.8. Statistical Analysis

Data were expressed as mean values (mean ± SD). The significance of differences between the two groups was evaluated with Mann–Whitney test. The significance of differences among the groups was evaluated using Kruskal–Wallis test, followed by Dunn’s multiple comparison post hoc test. Statistical analyses were performed using Prism 9.2 statistical software (GraphPad Software, La Jolla, CA, USA). A probability value <0.05 was considered statistically significant.

## 3. Results

### 3.1. Maternal Fructose Exposure Exacerbates Ventricular Pressure Overload-Induced Cardiac Hypertrophy in Adult Offspring

To determine whether MFE has an effect on the regulation of cardiac developmental programming and cardiac stress response in adulthood, we performed transverse aortic constriction (TAC) to induce ventricular pressure overload in 3-month-old offspring with or without MFE. Four weeks post-TAC, echocardiography was used to examine cardiac dimensions and ejection function (Table 1), followed by heart weight measurement, histopathological examination, and biochemical analysis. The results showed no significant impact of either TAC and MFE on the body weight of offspring, while the heart weight in offspring with TAC treatment was higher than that in those without. Of interest, a trend of heart weight increment was observed in offspring with MFE despite the lack of statistical significance (Table 1). Echocardiographic assessment further indicated that both interventricular septal thickness in diastole (IVS; d) and left ventricular posterior wall thickness in diastole (LVPW; s) were increased in offspring with concomitant MFE and TAC. On the other hand, although neither MFE nor TAC had a significant functional impact on left ventricular ejection fraction and stroke volume (Table 1), excessive maternal fructose intake during pregnancy and lactation still anatomically exacerbated TAC-induced cardiac hypertrophy.

### 3.2. Maternal Fructose Exposure Exacerbates Ventricular Pressure Overload-Induced Cardiac Remodeling in Adult Offspring

**Figure 1:**
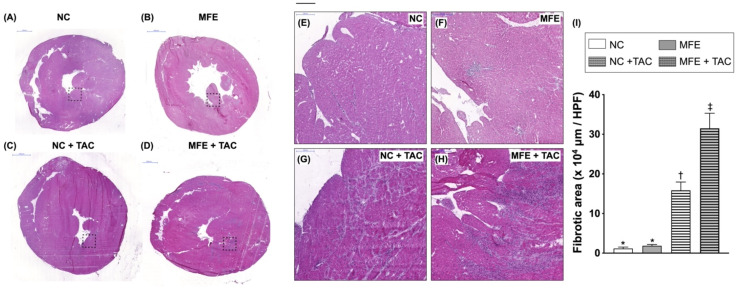
Hematoxylin and eosin (H&E) staining on myocardial sections of offspring with maternal fructose exposure (MFE) and ventricular pressure overload induced by transverse aortic constriction (TAC). (**A**,**E**) Normal control rat offspring. (**B**,**F**) Rat offspring with MFE. (**C**,**G**) Normal control rat offspring with TAC. (**D**,**H**) Rat offspring with MFE and TAC. (**I**) Comparison of myocardial infarcted areas among different treatment groups. (**E**–**H**) Enlarged image from the area within dotted rectangles in (**A**–**D**). NC, normal control. Groups with different symbols (*, †, ‡), *p* < 0.05. *n* = 8 for each group. Scale bars in left upper corner of (**A**–**D**) and (**E**–**H**) indicate 2000 and 200 μm, respectively.

To test whether MFE would exacerbate the impact of ventricular pressure overload-induced cardiac remodeling at histological level, we performed hematoxylin and eosin (H&E) staining on myocardial sections of offspring with or without MFE and TAC. Similar to that observed in echocardiography, ventricular hypertrophy was found in offspring with TAC on histopathologic examination (Figure 1). Moreover, an exacerbation of TAC-induced cardiac remodeling by MFE was reflected by a further increase in area of ventricular fibrosis compared to that in animals subjected to TAC alone (Figure 1). Of interest, under normal conditions, mild myocardial fibrosis was observed in offspring with MFE (Figure 1).

**Figure 2:**
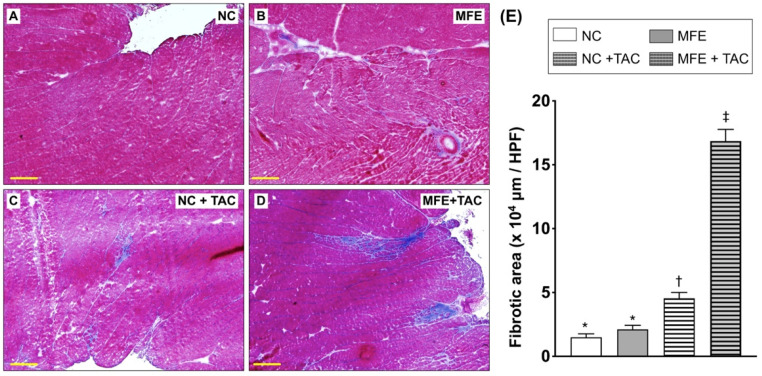
Masson’s trichrome (MTC) staining on myocardial sections of offspring with maternal fructose exposure (MFE) and ventricular pressure overload induced by transverse aortic constriction (TAC). (**A**) Normal control rat offspring. (**B**) Rat offspring with MFE. (**C**) Normal control rat offspring with TAC. (**D**) Rat offspring with MFE and TAC. (**E**) Comparison of myocardial fibrotic areas among treatment different groups. NC, normal control. Groups with different symbols (*, †, ‡), *p* < 0.05. *n* = 8 for each group. Scale bar in left lower corner indicates 200 μm.

**Figure 3:**
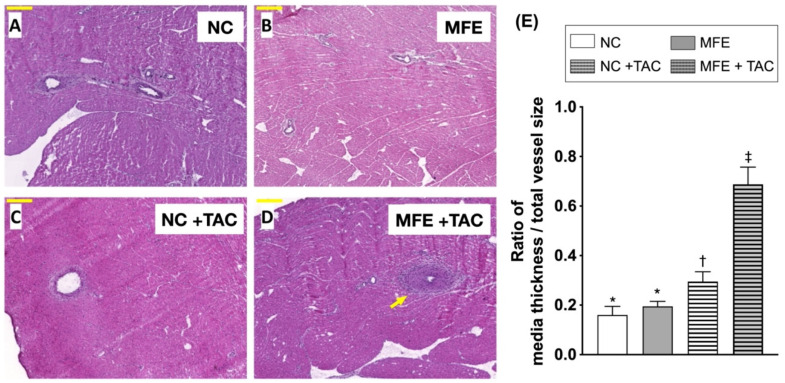
Histological quantification of myocardial arterial muscularization in offspring with maternal fructose exposure (MFE) and ventricular pressure overload induced by transverse aortic constriction (TAC) on sections with hematoxylin and eosin (H&E) staining. (**A**) Normal control rat offspring. (**B**) Rat offspring with MFE. (**C**) Normal control rat offspring with TAC. (**D**) Rat offspring with MFE and TAC. (**E**) Comparison of the degree of myocardial arterial muscularization in vessels with diameters > 100 μm among different treatment groups. The yellow arrow indicates myocardial arterial muscularization in offspring with MFE and TAC. NC, normal control. Groups with different symbols (*, †, ‡), *p* < 0.05. *n* = 8 for each group. Scale bar in left lower corner indicates 200 μm.

In addition to H&E staining, Masson’s trichrome (MTC) staining was used to quantitate myocardial collagen deposition. Consistent with the results of H&E staining, MFE further aggravated TAC-induced myocardial collagen deposition in offspring (Figure 2). Similar to that in H&E, myocardial collagen deposition was also observed only in offspring with MFE. Intriguingly, MFE enhanced arterial muscularization in the myocardium of offspring with TAC (Figure 3). Taken together, the aforementioned experimental results all suggest an adverse effect of excessive maternal fructose intake during pregnancy and lactation on cardiac development programing and ventricular pressure overload-triggered cardiac remodeling.

### 3.3. Maternal Fructose Exposure Increases Oxidative Stress in the Myocardium Subjected to Ventricular Pressure Overload

**Figure 4:**
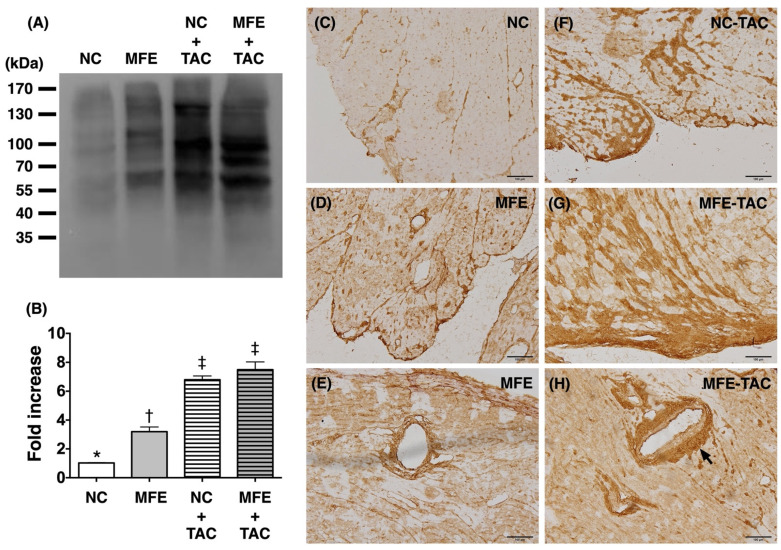
Elevated myocardial oxidative stress in offspring with maternal fructose exposure (MFE) and ventricular pressure overload induced by transverse aortic constriction (TAC). (**A**) OxyBlot showing expression levels of myocardial oxidized proteins as fold increase relative to NC group. (**B**) Comparison of myocardial oxidized protein expressions among different treatment groups. (**C**–**H**) OxyIHC demonstrating the distribution of myocardial oxidized proteins. Black arrow indicates significant accumulations of oxidized proteins in myocardial muscularized arteries of offspring with MFE and TAC. NC, normal control. Groups with different symbols (*, †, ‡), *p* < 0.05. *n* = 8 for each group. Scale bar in left lower corner indicates 100 μm.

Oxidative stress is identified as a contributor to the development of cardiac hypertrophy. To determine whether oxidative stress is involved in MFE-induced cardiac hypertrophy, we examined the level of myocardial oxidized protein which was found to be increased in offspring with MFE (Figure 4A,B). On the other hand, the marked elevation in the level of myocardial oxidized protein after TAC may have masked the impact of MFE, resulting in a nonsignificant contribution of MFE in this setting (Figure 4A,B). An investigation into the distribution of oxidized proteins in the myocardium of offspring after TAC revealed an elevated expression of oxidized proteins in the left periventricular areas and in arteries with significant muscularization (Figure 4C–H).

### 3.4. Maternal Fructose Exposure Regulates the Gene Expression Profiles and Stress Response Signaling in the Myocardium of Adult Offspring

**Figure 5:**
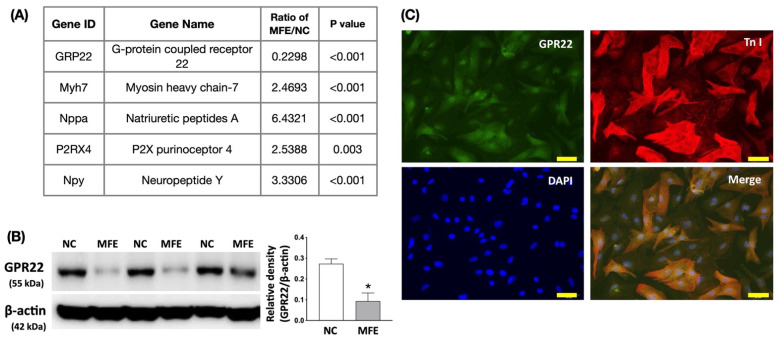
Maternal fructose exposure regulates gene expression levels of proteins involved in cardiac function and remolding. (**A**) The list of cardiac function-related genes with more than 2-fold changes in the myocardium of offspring with or without MFE. (**B**) Protein expression of GPR22 in the myocardium of offspring with or without MFE. (**C**) Immunofluorescent staining with antibodies against GPR22 and a cardiomyocyte marker, troponin I (Tn I), to examine the distribution of GPR22 in cardiac cells. NC, normal control. MFE, maternal fructose exposure. * indicates significance compared with NC group. Scale bar in right lower corner of (**C**) indicates 50 μm.

To investigate the mechanisms underlying MFE-induced cardiac developmental programming, next generation sequencing (NGS)-based RNA sequencing was used to analyze the gene expression profiles in the myocardium of 3-month-old offspring with or without MFE. Among genes whose transcription levels were up- or downregulated by MFE by more than 2-fold (Supplementary Table S1), several cardiac function-associated genes, including G protein-coupled receptor 22 (GPR22), myosin heavy chain-7 (Myh-7), atrial natriuretic peptide (Nppa/ANP), P2X purinoceptor 4 (P2RX4), and neuropeptide Y (Npy), were identified (Figure 5A). One of the identified genes, GPR22, was found to contribute to the progression of ventricular pressure overload-induced cardiac remodeling [21]. Western blotting consistently demonstrated a downregulation of GPR22 protein expression in offspring with MFE treatment (Figure 5B). Immunofluorescent staining on primary neonatal cardiac cells further indicated the specific expression of GPR22 in cardiomyocytes but not in other cellular elements (Figure 5C).

**Figure 6:**
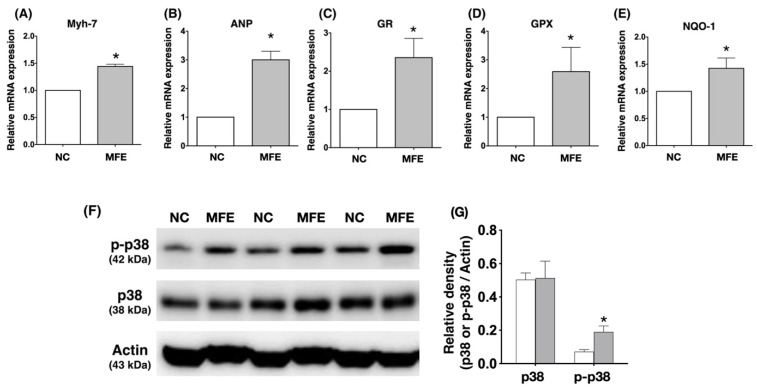
Activation of stress response signaling in the myocardium of offspring with maternal fructose exposure. (**A**) mRNA expression level of Myh-7. (**B**) mRNA expression level of ANP. (**C**) mRNA expression level of GR. (**D**) mRNA expression level of GPX in the myocardium. (**E**) mRNA expression level of NQO-1 in the myocardium. (**F**) Protein expression level of phosphorylated and total p38-MAPK in the myocardium. (**G**) Comparisons of the ratio of phosphorylated to total p38-MAPK expression in the myocardium of offspring. NC, normal control. MFE, maternal fructose exposure. TAC, transverse aortic constriction. Scale bar in right lower corner of (**C**) indicates 50 μm. NC, normal control. MFE, maternal fructose exposure. Myh-7, myosin heavy chain-7. ANP, atrial natriuretic peptide. GR, Glutathione reductase. GPX, Glutathione peroxidase. NQO-1, NAD(P)H dehydrogenase quinone 1. * indicates significance compared with NC group.

In addition to NGS, we also adopted real-time quantitative PCR (RT-qPCR) to determine the mRNA expression of cardiac stress response genes in the myocardium of offspring. In concert with the cardiac hypertrophy observed in echocardiography and histopathological examination, RT-qPCR showed higher myocardial mRNA expression levels of Myh7 and ANP, indices of cardiac hypertrophy and pressure overload, in offspring with MFE than those in offspring without (Figure 6A,B). Since MFE was found to contribute to oxidative stress in the myocardium, the expression levels of anti-oxidant genes, whose expressions are triggered by oxidative stress, were also examined with RT-qPCR. The results showed an increase in transcripts of anti-oxidant genes, such as GR, GPx, and NQO-1, in the myocardium of offspring with MFE (Figure 6C–E). Among the signaling pathways involved in cardiac hypertrophy, p38-MAPK plays a controversial role in cardiac remodeling and could be activated by oxidative stress [22,23,24]. Western blots showed a significant increase in the myocardial levels of phosphorylated p38 in offspring with MFE (Figure 6E).

## 4. Discussion

Although the effect of maternal fructose intake on developmental programming has been revealed in recent decades, its cardiac influence remains unknown. In this study, we first demonstrated that excessive excessive maternalintake of fructose during pregnancy and lactation resulted in mild myocardial hypertrophy (Table 1) and exacerbated cardiac remodeling post TAC-induced ventricular pressure overload (Figure 1). In addition to fibrosis (Figure 2), arterial muscularization (Figure 3) and an increase in oxidative stress (Figure 4) were observed in the myocardium of offspring with MFE. We then performed NGS-based mRNA sequencing to analyze myocardial gene expression profiles and found that an orphan G protein-coupled receptor, GPR-22, was associated with maternal fructose intake-induced cardiac developmental programming (Figure 5). The upregulation of stress response proteins and the activation of p38-MAPK were also observed in the myocardium of offspring with MFE (Figure 6).

Our recent study indicated that the function of endothelial progenitor cells (EPC) and endothelial cells of offspring is impaired by excessive maternal fructose intake [20]. Previous studies also demonstrated that maternal fructose intake is involved in the developmental programming of the renal system and results in hypertension in rat models [18,25]. Hypertension and endothelial dysfunction are considered to be key factors in the initiation and progression of myocardial diseases, such as cardiac hypertrophy and heart failure [26,27,28]. In this study, we further investigated whether maternal fructose intake during pregnancy and lactation modulates cardiac developmental programming and aggravates the progression of ventricular pressure overload-induced cardiac remodeling. Echoing the finding of MFE-induced hypertension in adult offspring [18], mild cardiac hypertrophy, myocardial fibrosis, and collagen deposition were all noted in offspring without TAC-induced ventricular pressure overload (Figure 1 and Figure 2). Furthermore, coronary arterial remodeling, which is reported to be induced by ventricular pressure overload [29], was also exacerbated by MFE in the current study (Figure 3) as reflected by a significant increase in thickness of the arterial media layer in the myocardium of offspring with MFE and TAC. Besides, elevated oxidative stress was also observed in the muscularized coronary artery (Figure 4). The co-occurrence of arterial muscularization and the increment of oxidative stress in the myocardial artery echoes the finding of a previous study, in which an oxidative stress-induced proliferation of vascular smooth muscle cells was reported [30]. The aforementioned investigations all imply that MFE-induced cardiac remodeling might be related to renal developmental programming and endothelial dysfunction, which have been reported to be modulated by MFE [18,20,25].

Oxidative stress is considered to be a critical contributor to cardiac hypertrophy [31]. Previous studies have demonstrated an increased oxidative stress associated with fructose treatment in cardiomyocytes as well as cardiac hypertrophy in animals with excessive fructose intake [32,33]. However, whether excessive maternal fructose intake during pregnancy and lactation also causes an increase in oxidative stress in the myocardium of offspring remains unknown. In the present study, elevations in myocardial oxidative stress and the gene expression level of anti-oxidant proteins in offspring with MFE treatment implied that an MFE-associated increase in myocardial oxidative stress may contribute to cardiac hypertrophy in adult offspring. Previous studies have indicated that oxidative stress could activate cardiac hypertrophy-related signal transductions, such as inflammatory cytokines and MAPK pathways [31]. In this study, we also found an MFE-related upregulation of p38-MAPK activation in the myocardium (Figure 6). Hence, in addition to possible influences from renal and endothelial systems, myocardial oxidative stress and activation of p38-MAPK may also play important roles in MFE-induced cardiac hypertrophy.

Through RNA sequencing, mRNA expressions of hundreds of genes were found to be regulated by MFE (Supplementary Table S1). However, only a few genes, such as Myh-7, GPR22, Nppa, P2RX4, and Npy, have been reported to be directly involved in cardiac remodeling and myocardial stress response [21,34,35,36,37]. Myh-7 and Nppa are wildly used as markers for cardiac hypertrophy and pressure overload [37], while P2RX4 is considered to play a beneficial role in alleviating cardiac hypertrophy and heart failure in a canine calsequestrin (CSQ) transgenic mouse model [36]. Although Npy was originally regarded as a neuropeptide, the role of NPY in inducing cardiac hypertrophy has been reported in recent studies [34,35,38]. GPR22, a G protein-coupled receptor, whose myocardial expression is regulated by the induction of diabetes in rats [39]. In the present study, both mRNA and protein levels of GPR22 were dramatically reduced by MFE (Figure 4). In a mouse model with GPR22 gene disruption, an increased susceptibility to functional decompensation following TAC was observed [21]. It is reasonable that cardiac remodeling exacerbated by MFE may be mediated by the downregulation of myocardial GPR22 expression. However, further studies are needed to clarify the mechanisms by which MFE induces cardiac developmental programing in offspring.

There are some limitations in this study. Despite a significant exacerbation of TAC-induced cardiac remodeling in offspring with MFE, the lack of significant difference in contractile function compared with their littermates without MFE may be due to the limited number of animals as well as individual variations in the amount of maternal fructose intake and responses to ventricular pressure overload. In addition, a long-term observation is needed to further elucidate the impact of MFE on cardiac function in the offspring in late adulthood. Furthermore, although the findings of this study demonstrate a regulatory role of MFE in cardiac myocardial gene expression and stress response to ventricular pressure overload, further investigations are needed to reveal the mechanistic interactions among maternal fructose intake, cardiac developmental programming, and response to myocardial stress in offspring.

## 5. Conclusions

Through this study, we found that excessive maternal fructose intake during pregnancy and lactation increased myocardial oxidative stress and led to mild cardiac hypertrophy. In addition, high maternal fructose intake also exacerbated the cardiac remodeling induced by ventricular pressure overload. However, whether fructose-derived metabolites participate in cardiac developmental programming in offspring remains unknown. In addition, the role of GPR22 in MFE-induced cardiac hypertrophy is still unclear and needs to be further investigated.

## Figures and Tables

**Table 1:** Body weight, heart weight, and echocardiographic characteristics of offspring with maternal fructose exposure and ventricular pressure overload.

	NC	MFE	NC + TAC ^a^	MFE + TAC	*p* Value
Body weight ^b^ (g)	628.88 ± 59.84	625.88 ± 25.98	637.88 ± 43.48	650.25 ± 49.42	0.7391
Heart weight ^b^ (g)	1.449 ± 0.196	1.604 ± 0.099	1.751 ± 0.118 *	1.818 ± 0.186 *^,†^	<0.005
Echocardiography ^b^					
IVS; d (mm)	1.492 ± 0.232	1.513 ± 0.123	1.552 ± 0.225	1.646 ± 0.149	0.4051
IVS; s (mm)	2.163 ± 0.271	2.257 ± 0.256	2.316 ± 0.132	2.539 ± 0.333 *	0.0439
LVPW; d (mm)	1.858 ± 0.172	1.849 ± 0.142	2.057 ± 0.786	2.175 ± 1.117	0.9890
LVPW; s (mm)	2.422 ± 0.255	2.61 ± 0.297	2.645 ± 0.368 *	2.963 ± 0.224 *^,†^	0.0058
LVID; d (mm)	8.899 ± 0.628	8.935 ± 0.302	9.111 ± 0.57	9.346 ± 0.443	0.1219
LVID; s (mm)	5.89 ± 0.684	5.749 ± 0.686	5.796 ± 0.482	5.707 ± 0.692	0.8478
LVEF (%)	60.37 ± 6.57	62.53 ± 8.79	62.40 ± 5.52	63.96 ± 7.84	0.8847
SV (μL)	301.3 ± 36.24	310.5 ± 55.14	311.8 ± 52.25	321.0 ± 52.95	0.8634

^a^ Ventricular pressure overload induced by transverse aortic constriction (TAC) in 3-month-old male offspring. ^b^ Heart and body weight measurement as well as ultrasonography for cardiac dimensional and functional assessment in offspring with or without maternal fructose exposure (MFE) at four weeks post-TAC. NC, normal control; IVS; d, Interventricular septal thickness in diastole; IVS; s, Interventricular septal thickness in systole; LVPW; d, left ventricular posterior wall thickness in diastole; LVPW; s, left ventricular posterior wall thickness in diastole; LVID; d, left ventricular internal dimension in diastole; LVID; s, left ventricular internal dimension in systole; LVEF, left ventricle ejection fraction; SV, stroke volume (SV). * *p* < 0.05 compared with NC group, ^†^ *p* < 0.05 compared with MFE group, *n* = 8 for each group.

## Supplementary Materials

The following are available online at https://www.mdpi.com/article/10.3390/nu13093267/s1, Table S1: Myocardial gene expression profile of 3-month-old offspring.

## References

[1] Bray G.A., Nielsen S.J., Popkin B.M. (2004). Consumption of high-fructose corn syrup in beverages may play a role in the epidemic of obesity. Am. J. Clin. Nutr..

[2] Bantle J.P. (2009). Dietary fructose and metabolic syndrome and diabetes. J. Nutr..

[3] Tappy L., Le K.A., Tran C., Paquot N. (2010). Fructose and metabolic diseases: New findings, new questions. Nutrition.

[4] Hwang I.S., Ho H., Hoffman B.B., Reaven G.M. (1987). Fructose-induced insulin resistance and hypertension in rats. Hypertension.

[5] Elliott S.S., Keim N.L., Stern J.S., Teff K., Havel P.J. (2002). Fructose, weight gain, and the insulin resistance syndrome. Am. J. Clin. Nutr..

[6] Deng J.Y., Huang J.P., Lu L.S., Hung L.M. (2007). Impairment of cardiac insulin signaling and myocardial contractile performance in high-cholesterol/fructose-fed rats. Am. J. Physiol. Heart Circ. Physiol..

[7] Sanchez-Lozada L.G., Tapia E., Jimenez A., Bautista P., Cristobal M., Nepomuceno T., Soto V., Avila-Casado C., Nakagawa T., Johnson R.J. (2007). Fructose-induced metabolic syndrome is associated with glomerular hypertension and renal microvascular damage in rats. Am. J. Physiol. Renal. Physiol..

[8] Miller A., Adeli K. (2008). Dietary fructose and the metabolic syndrome. Curr. Opin. Gastroenterol..

[9] Lucas A. (1991). Programming by early nutrition in man. Ciba Found. Symp..

[10] Williams S.J., Campbell M.E., McMillen I.C., Davidge S.T. (2005). Differential effects of maternal hypoxia or nutrient restriction on carotid and femoral vascular function in neonatal rats. Am. J. Physiol. Regul. Integr. Comp. Physiol..

[11] Li G., Xiao Y., Estrella J.L., Ducsay C.A., Gilbert R.D., Zhang L. (2003). Effect of fetal hypoxia on heart susceptibility to ischemia and reperfusion injury in the adult rat. J. Soc. Gynecol. Investig..

[12] Bae S., Xiao Y., Li G., Casiano C.A., Zhang L. (2003). Effect of maternal chronic hypoxic exposure during gestation on apoptosis in fetal rat heart. Am. J. Physiol. Heart Circ. Physiol..

[13] Battista M.C., Calvo E., Chorvatova A., Comte B., Corbeil J., Brochu M. (2005). Intra-uterine growth restriction and the programming of left ventricular remodelling in female rats. J. Physiol..

[14] Han H.C., Austin K.J., Nathanielsz P.W., Ford S.P., Nijland M.J., Hansen T.R. (2004). Maternal nutrient restriction alters gene expression in the ovine fetal heart. J. Physiol..

[15] Wang J., Ma H., Tong C., Zhang H., Lawlis G.B., Li Y., Zang M., Ren J., Nijland M.J., Ford S.P. (2010). Overnutrition and maternal obesity in sheep pregnancy alter the JNK-IRS-1 signaling cascades and cardiac function in the fetal heart. FASEB J..

[16] Huang Y., Yan X., Zhao J.X., Zhu M.J., McCormick R.J., Ford S.P., Nathanielsz P.W., Ren J., Du M. (2010). Maternal obesity induces fibrosis in fetal myocardium of sheep. Am. J. Physiol. Endocrinol. Metab..

[17] Dong M., Zheng Q., Ford S.P., Nathanielsz P.W., Ren J. (2012). Maternal obesity, lipotoxicity and cardiovascular diseases in offspring. J. Mol. Cell Cardiol..

[18] Tain Y.L., Leu S., Wu K.L., Lee W.C., Chan J.Y. (2014). Melatonin prevents maternal fructose intake-induced programmed hypertension in the offspring: Roles of nitric oxide and arachidonic acid metabolites. J. Pineal. Res..

[19] Tain Y.L., Wu K.L., Lee W.C., Leu S., Chan J.Y. (2015). Maternal fructose-intake-induced renal programming in adult male offspring. J. Nutr. Biochem..

[20] Leu S., Wu K.L.H., Lee W.C., Tain Y.L., Chan J.Y.H. (2019). The Impact of Maternal Fructose Exposure on Angiogenic Activity of Endothelial Progenitor Cells and Blood Flow Recovery after Critical Limb Ischemia in Rat Offspring. Int. J. Mol. Sci..

[21] Adams J.W., Wang J., Davis J.R., Liaw C., Gaidarov I., Gatlin J., Dalton N.D., Gu Y., Ross J., Behan D. (2008). Myocardial expression, signaling, and function of GPR22: A protective role for an orphan G protein-coupled receptor. Am. J. Physiol. Heart Circ. Physiol..

[22] Zhang S., Weinheimer C., Courtois M., Kovacs A., Zhang C.E., Cheng A.M., Wang Y., Muslin A.J. (2003). The role of the Grb2-p38 MAPK signaling pathway in cardiac hypertrophy and fibrosis. J. Clin. Investig..

[23] Braz J.C., Bueno O.F., Liang Q., Wilkins B.J., Dai Y.S., Parsons S., Braunwart J., Glascock B.J., Klevitsky R., Kimball T.F. (2003). Targeted inhibition of p38 MAPK promotes hypertrophic cardiomyopathy through upregulation of calcineurin-NFAT signaling. J. Clin. Investig..

[24] Liang Q., Molkentin J.D. (2003). Redefining the roles of p38 and JNK signaling in cardiac hypertrophy: Dichotomy between cultured myocytes and animal models. J. Mol. Cell Cardiol..

[25] Elshenawy S., Simmons R. (2016). Maternal obesity and prenatal programming. Mol. Cell Endocrinol..

[26] Yildiz M., Oktay A.A., Stewart M.H., Milani R.V., Ventura H.O., Lavie C.J. (2020). Left ventricular hypertrophy and hypertension. Prog. Cardiovasc. Dis..

[27] Iliev A., Kotov G., Dimitrova I.N., Landzhov B. (2019). Hypertension-induced changes in the rat myocardium during the development of cardiac hypertrophy—A comparison between the left and the right ventricle. Acta Histochem..

[28] Boulanger C.M. (1999). Secondary endothelial dysfunction: Hypertension and heart failure. J. Mol. Cell Cardiol..

[29] Yang F., Dong A., Mueller P., Caicedo J., Sutton A.M., Odetunde J., Barrick C.J., Klyachkin Y.M., Abdel-Latif A., Smyth S.S. (2012). Coronary artery remodeling in a model of left ventricular pressure overload is influenced by platelets and inflammatory cells. PLoS ONE.

[30] Satoh K., Nigro P., Berk B.C. (2010). Oxidative stress and vascular smooth muscle cell growth: A mechanistic linkage by cyclophilin A. Antioxid. Redox Signal..

[31] Rababa’h A.M., Guillory A.N., Mustafa R., Hijjawi T. (2018). Oxidative Stress and Cardiac Remodeling: An Updated Edge. Curr. Cardiol. Rev..

[32] Park J.H., Ku H.J., Kim J.K., Park J.W., Lee J.H. (2018). Amelioration of High Fructose-Induced Cardiac Hypertrophy by Naringin. Sci. Rep..

[33] Zhang Y.B., Meng Y.H., Chang S., Zhang R.Y., Shi C. (2016). High fructose causes cardiac hypertrophy via mitochondrial signaling pathway. Am. J. Transl. Res..

[34] Xie Y., Hu J., Zhang X., Li C., Zuo Y., Xie S., Zhang Z., Zhu S. (2020). Neuropeptide Y Induces Cardiomyocyte Hypertrophy via Attenuating miR-29a-3p in Neonatal Rat Cardiomyocytes. Protein Pept. Lett..

[35] McDermott B.J., Bell D. (2007). NPY and cardiac diseases. Curr. Top. Med. Chem..

[36] Yang A., Sonin D., Jones L., Barry W.H., Liang B.T. (2004). A beneficial role of cardiac P2X4 receptors in heart failure: Rescue of the calsequestrin overexpression model of cardiomyopathy. Am. J. Physiol. Heart Circ. Physiol..

[37] Cox E.J., Marsh S.A. (2014). A systematic review of fetal genes as biomarkers of cardiac hypertrophy in rodent models of diabetes. PLoS ONE.

[38] Wang J., Hao D., Zeng L., Zhang Q., Huang W. (2021). Neuropeptide Y mediates cardiac hypertrophy through microRNA-216b/FoxO4 signaling pathway. Int. J. Med. Sci..

[39] Ruiz-Hernandez A., Sanchez-Munoz F., Rodriguez J., Calderon-Zamora L., Romero-Nava R., Huang F., Hong E., Villafana S. (2015). Expression of orphan receptors GPR22 and GPR162 in streptozotocin-induced diabetic rats. J. Recept. Signal Transduct. Res..

